# Dynamic sustained attention markers differentiate atypical development: The case of Williams syndrome and Down's syndrome

**DOI:** 10.1016/j.neuropsychologia.2019.107148

**Published:** 2019-09

**Authors:** Nir Shalev, Ann Steele, Anna C. Nobre, Annette Karmiloff-Smith, Kim Cornish, Gaia Scerif

**Affiliations:** a Department of Experimental Psychology, University of Oxford, United Kingdom; b University of Manchester, United Kingdom; c Oxford Centre for Human Brain Activity, Department of Psychiatry, Wellcome Centre for Integrative Neuroimaging, University of Oxford, United Kingdom; d Birkbeck, University of London, United Kingdom; e School of Psychological Sciences, Monash University, Australia

## Abstract

Impaired sustained attention is considered an important factor in determining poor functional outcomes across multiple cognitive and behavioural disorders. Sustained attention is compromised for both children with Williams syndrome (WS) and Down's syndrome (DS), but specific difficulties remain poorly understood because of limitations in how sustained attention has been assessed thus far.

In the current study, we compared the performance of typically developing children (N = 99), children with WS (N = 25), and children with DS (N = 18), on a Continuous Performance Task – a standard tool for measuring sustained attention. In contrast to previous studies, primarily focused on overall differences in mean performance, we estimated the extent to which performance changed over time on task, thus focusing directly on the *sustained* element of performance. Children with WS and children with DS performed more poorly overall compared to typically developing children. Importantly, measures specific to changes over time differentiated between children with the two syndromes. Children with WS showed a decrement in performance, whereas children with Down's syndrome demonstrated non-specific poor performance. In addition, our measure of change in performance predicted teacher-rated attention deficits symptoms across the full sample.

An approach that captures dynamic changes in performance over assessments may be fruitful for investigating similarities and differences in sustained attention for other atypically developing populations.

## Highlights

• Impaired sustained attention is a key factor in determining poor functional outcomes.
• Children with WS or DS performed more poorly overall than neurotypical children.
• In addition, children with WS showed a decrement in performance over time.
• In contrast, children with DS demonstrated non-specific poor performance.
• Our dynamic sustained attention measure predicted teacher-rated attention symptoms.

## 1 Introduction

Sustained attention is thought to be fundamental in supporting higher-order cognitive functions (e.g., Sarter et al., 2001). As such, it is an important factor in typical development (e.g., Colombo, 2001; Scerif, 2010), helping the child acquire and shape domain-specific skills like numeracy (Steele et al., 2012). In adolescents, lower performance in sustained attention tasks is correlated with poor outcomes in learning and is associated with behavioural and emotional difficulties (e.g., Shalev et al., 2015). Difficulties in sustaining attention have been linked to multiple acquired and neurodevelopmental disorders. These include Attention Deficit Hyperactivity Disorder (“ADHD”, Barkley, 1997), autism (Garretson et al., 1990), Learning Difficulties (Richards et al., 1990), schizophrenia and affective disorders (Liu et al., 2002), bipolar disorder (Clark et al., 2002), as well as chronic stroke (Hyndman and Ashburn, 2003), traumatic brain injuries (Robertson et al., 1997), and more.

Poor sustained attention has also been associated with atypical development in genetically diagnosed conditions, such as Williams syndrome and Down's syndrome. Williams syndrome arises from the deletion of some 28 genes on chromosome 7 (Donnai and Karmiloff-Smith, 2000), and Down's syndrome is caused by trisomy of chromosome 21 (Antonarakis et al., 2004). Williams syndrome is characterised by poor visuo-spatial abilities alongside relatively preserved language skills, whereas Down's syndrome is characterised by lower language skills alongside less impaired visuo-spatial skills (e.g. Mervis and John, 2012). At the behavioural level, both groups are reported to be more inattentive, hyperactive, and distractible than neuro-typical children according to parent or teacher reports or structured clinical interviews (Dodd and Porter, 2009; Porter et al., 2008). These reports are supported by a high co-morbidity of ADHD with Down's (Ekstein et al., 2011) and Williams syndrome (Leyfer et al., 2006).

Researchers have used cognitive tasks to measure and describe difficulties in sustained attention among children with Down's syndrome or Williams syndrome (Costanzo et al., 2013; Menghini et al., 2010; Rowe et al., 2006). A close inspection of these empirical findings reveals that some of the reports of poor sustained attention may be conflicting. The following section will present a brief overview of the evidence for sustained attention impairments within Down's syndrome and Williams syndrome, focusing in particular on important measurement issues characterising previous studies.

### 1.1 Measuring sustained attention in atypically developing children

The Continuous Performance Task (CPT) is commonly used to characterise sustained attention in neurotypical adults (Conners and Staff, 2000; Cornblatt et al., 1988; Greenberg, 1987; Klee and Garfinkel, 1983; Lee and Park, 2006; Robertson et al., 1997; Rosvold et al., 1956; Shalev et al., 2016). On a CPT task, individuals are presented with a serial stream of stimuli which appear at the centre of the screen. Within the stream there are ‘targets’, to which participants are requested to respond; and ‘distractors’ that should be ignored. One of the unique characters of a CPT is its continuous nature: the serial presentation of stimuli is independent of responses. Importantly, one of the main challenges on most CPTs is the requirement to stay engaged to a simple, repetitive and non-engaging continuous task for a relatively long time. Although it is widely used, the CPT has multiple limitations when applied to clinical populations, including the feasibility of performing prolonged tasks and the problem of identifying meaningful performance markers. Typically, individual performance is assessed based on reaction times (RTs) to targets or number of errors (either missing targets or responding to distractors). Traditional approaches, influenced by the notion of ‘vigilance’, emphasise performance decrement as the variable of interest (e.g., Parasuraman, 1979). This approach may be inadequate in a clinical context, where assessment is required to be relatively short and therefore a reliable decrement may not be observed. Another way to measure sustained attention in a relatively short time is to estimate the fluctuations of performance over time by measuring the standard deviation of RTs during the task (e.g. Shalev et al., 2011). RT-based indices can, however, be unreliable when assessing clinical populations because of limited trial numbers and possible confounding motor difficulties. In accordance with these limitations, researchers investigating sustained attention in young or atypically developing children have often used discrete outcome measures based on overall accuracy during a task rather than speeded responses, and without focusing on performance decrements over time (e.g., Breckenridge et al., 2013).

While relying on accuracy-based measures from brief tasks seems intuitive for these populations, it is still necessary to ensure that the relevant aspect of cognition (i.e., performance maintenance over time) is appropriately captured. For example, Costanzo et al. (2013) reported unimpaired visual sustained attention among children with Down's and Williams syndromes, but this skill was assessed based on performance on a cancellation task primarily aimed at measuring spatial attention. Whereas sustained attention may undeniably influence performance on any cognitive task, cancellation tasks do not strictly require sustained attention (e.g., Robertson et al., 1997), and provides a poor measure of sustained attention when contrasted with a CPT (e.g., Oades, 2000). In contrast with their reports of unimpaired visual sustained attention, Costanzo et al. (2013) identified poor performance in an auditory counting task which was intended to estimate auditory sustained attention. Nonetheless, as with the cancellation task, the authors only focused on overall mean accuracy and did not account for the way in which performance may have changed over time.

The same method to assessing sustained attention based on a visual cancellation task and an auditory counting task was applied by Menghini et al. (2010) to children with Williams syndrome, reporting findings conflicting with those of Costanzo et al. (2013): poor sustained attention in both the visual and auditory modalities. Rowe, Lavender and Turk (2006) studied a group of children with Down's syndrome and argued for a sustained attention deficit based on a visual search task in which participants searched for a pre-defined target presented among distractors. Arguably, as with the cancellation task that was used by Costanzo et al. (2013), this approach may not tax sustained attention clearly as it relies on the ability to use selective attention to identify goal-related targets amongst competing distractors over a short period of time.

In another case, Brown et al. (2003) assessed sustained attention in toddlers with Williams syndrome and Down's syndrome by measuring how long the toddlers maintained their interest in playing with study toys. They reported relatively preserved performance among the Williams syndrome children, and poor performance among the children with Down's syndrome. While this approach for measuring sustained attention is common practice when studying toddlers, the play context differs significantly from classic sustained attention tasks, which are characterised by being repetitive and non-engaging. Additional factors in the play context, such a novelty and arousal may therefore mask effects specifically related to sustaining attention. In contrast with Brown et al. (2003), some evidence suggests that sustained attention is affected in children with WS. Mervis et al. (2003) and Atkinson et al. (2003) found an unusual pattern of ‘sticky fixation’ (a term indicating an intense fixation on specific visual objects in the visual field, which interferes with the ability to process environmental changes). Such a behavioural pattern is thought to be a predictor of atypical attention maintenance (Cornish and Wilding, 2010).

More recent studies have applied more traditional, CPT approaches to measure sustained attention, but unfortunately results are also contradictory. Breckenridge et al. (2013) used both auditory and visual variations of CPTs and found no significant differences between children with WS, DS, and a control group matched for mental age. In the same study, they also reported a relative strength in the auditory sustained attention task among individuals with DS (Breckenridge et al., 2013). In another study using a CPT (alongside an extensive neuropsychological assessment battery), children with DS were compared to a group of children diagnosed with Fragile-X syndrome and a control group, subdivided into poor and good attenders (Munir et al., 2000). Although children with DS performed worse than the control group on some of the CPT outcome measures (e.g., they had a higher number of false alarms), they performed significantly better than children with Fragile-X syndrome and comparably better than the neurotypical control children who were identified as having poor attention.

One possible explanation for the inconclusive evidence concerning sustained attention is that the available studies all fail to incorporate an important aspect: the capacity to *sustain* attention over time (‘vigilance decrement’ effects; e.g., Parasuraman et al., 1998). When evaluated in detail, none of the studies above measured how performance changed over time. The main motivation of the current experimental investigation is to offer an additional perspective on sustained attention for children with Down's and Williams syndromes, by incorporating more dynamic measures accounting for performance change. Even though the existing findings are notably conflicting, we can (carefully) hypothesize that a selective attention impairment is more likely to appear in the case of WS. Such speculation is based on the previous studies that have used continuous tasks, where children with DS often did not exhibit a specific difficulty in performance, and often outperformed other clinical groups (Breckenridge et al., 2013; Munir et al., 2000). In contrast, children with WS who were assessed using cognitive tasks have shown behavioural patterns that are associated with a sustained attention impairment (Atkinson et al., 2003; Cornish and Wilding, 2010), although direct evidence for vigilant decrement is missing.

### 1.2 Current study

The available evidence for a specific sustained attention deficit in either Down's syndrome or Williams syndrome from the CPT literature is inconclusive (e.g., Breckenridge et al., 2013). The current study focuses on performance maintenance, rather than overall mean differences, for children with Williams syndrome, Down's syndrome and neurotypical children. The primary measure of sustained attention considered is the change in performance between the two halves of a standard CPT which was previously validated with young neurotypical individuals (Steele et al., 2012). We used this performance-change index to characterise group differences and to predict ADHD symptoms as a behavioural reflection of poor classroom based sustained attention. In addition, we analysed the continuous trend of change in performance over time based on the full time-series of mean accuracy as a function of time on task, separately for each group.

Based on the available evidence, it was hypothesised that 1) children with Williams syndrome are likely to suffer from a sustained attention deficit, 2) children with Down's syndrome are more likely to show overall poor performance when compared with the control group, and 3) the performance-change index will be correlated with reports of ADHD symptoms across the whole sample.

## 2 Methods

Some of the neurotypical attention data (N = 83, accuracy and reaction time data) were used previously in a study examining attention as a predictor of educational outcomes in neurotypical children (Steele et al., 2012). Background demographic variables were also presented in a separate study examining reading skills in Williams and Down's syndromes (Steele et al., 2013). Importantly, the analysis approach and specific comparisons presented here have never been applied to these sustained attention data.

### 2.1 Participants

All experimental protocols were reviewed and approved by the Central University Research Ethics Committee of the University of Oxford. The neurotypical sample included 103 children aged 3–7, evenly distributed across age and gender. They were recruited from four local state primary schools and three local nurseries in the UK, following procedures set by the relevant research ethics review board. Children were recruited using an opt-in procedure. Following the school's agreement to participate, information letters with consent slips were sent to the parents of children in relevant age groups. Only children who returned a signed consent were included in the study. None of the children had a diagnosed learning disability or a diagnosed attention disorder.

Children with Down's syndrome were recruited through local support groups including the Down's Heart Group, South Bucks Down's Syndrome Group, and the Swindon Down's Group. Children with Williams syndrome were all recruited through the Williams Syndrome Foundation. The charity organisations posted information sheets and consent forms to all children on their databases between the ages of 4–8. Letters of consent were received back from 27 parents of children in each group. However, one child with Down's syndrome was excluded from the study for having mosaic Down's syndrome.^2^ Parents of participating children reported that none of the children in these two groups have received a formal diagnosis of attention deficit hyperactivity disorder by this point and that none of the children received psychostimulant medication.

Seven children with Down's syndrome and one with Williams syndrome did not complete the sustained attention task. Children who did not complete were not overall younger than completing children, but tended to have higher teacher-reported inattention and hyperactivity. In all, 19 children with Down's syndrome, age 4–8, and 26 children with Williams syndrome, age 5–8, participated to the study. In addition, participants who did not provide any response during the task (i.e., omitted all targets and did not commit any false alarms) were excluded, as this suggested that participants did not engage in the task. This exclusion affected 3 neuro-typical children (leaving a final sample of N = 99 contributing to the current study), 1 child with WS and 1 with DS (leaving N = 25 and N = 18 respectively).

The typically developing group was divided into three sub-groups based on chronological age in months. Splitting the data in this way allowed comparing five groups with a similar sample size, while providing chronological age- and ability-matched samples for children with Down's syndrome and Williams syndrome. Based on a series of t-tests corrected for multiple comparisons, we could verify that: 1) the groups of children with DS, WS and the older NT group did not differ in their mean chronological age; 2) Children with WS and DS were older than the younger and mid-NT groups; 3) All the NT groups differed in their mean age, verbal and non-verbal mental age; 4) Children with WS did not differ in their verbal mental age from younger and mid NT; 5) Children with DS did not differ from younger NT in their verbal and non-verbal mental age; 6) Children with WS and children with DS did not differed in their non-verbal mental age. Sample size, mean chronological age in months, non-verbal and verbal ability expressed as mental age equivalent in months for each experimental group appear in Table 1.

**Table 1:** Mean chronological age (months), sample size, verbal and non-verbal mental age (months) for each experimental group. Figures after the semicolon indicate standard deviation of the mean.

	NT-younger	NT-middle	NT-older	DS	WS
N	31	34	34	18	25
Chronological Age (“CA”)	45.63; 6.59	66.06; 6.31	85.52; 5.11	86.39; 13.17	79.44; 11.06
Verbal Mental Age (“VMA”)	51.29; 12.26	71.41; 14.93	89.59; 17.37	42.33; 9.57	63.08; 19.37
Non-verbal Mental age (“NVMA”)	49.39; 10.15	62.85; 10.83	88.18; 19.15	40.00; 9.37	38.56; 6.82

#### 2.1.1 Apparatus

Computerised tasks were presented on an Elo AccuTouch 17” touchscreen monitor using EPrime software. Responses were recorded using the RB-530 Cedrus response box. All children were asked to rest their index finger at a set position at the beginning of every trial and, if necessary, children across all groups were reminded to do so throughout, but in a way that aimed not interfere with performance unduly. They were requested to press the response button whenever identifying a pre-defined target which appeared among non-targets (see details below).

#### 2.1.2 CPT

Children were instructed to press a response button when identifying a pre-defined target within a stream of targets and non-targets. The targets were animal drawings, and the non-targets were drawings of everyday objects, chosen from the Snodgrass and Vanderwart image pool (Rossion and Pourtois, 2004). The testing session began with a short practice block with extended stimulus exposure time, and children were instructed on how to perform the task using verbal instruction and visual aids. During the experimental run, stimulus exposure time was set for 300 ms and followed by a fixed inter-stimulus interval of 1250 ms, in which a blank screen was presented (stimulus exposure duration and inter-stimulus intervals were chosen following piloting across the age range). A correct ‘hit’ to a target stimulus resulted in a ‘woohoo’ reward sound. No sound accompanied a ‘miss’ response. Incorrect responses following distractor stimuli resulted in a tone indicating an incorrect response. All feedback sounds lasted 450 ms. The task lasted approximately 4 min. One hundred stimulus trials were presented in a random order. Twenty percent contained targets (animals). Overall performance was estimated using: the percentage of correct responses, the percentage of omitted targets, and the percentage of false alarms. Change in performance, representing capacity to sustain attention, was based on the percentage change in accuracy between the first and second halves of the task. The main and novel goal of the current analysis was to compare profiles in sustained attention deficits using a putatively more sensitive performance variable that focuses on performance change, in contrast with designs that are commonly used among children.

#### 2.1.3 Procedure

All children were tested individually on the computerised CPT. Verbal and non-verbal abilities, as well as ADHD symptoms in the classroom, were also recorded using standardised tests and questionnaires (see details below).

#### 2.1.4 Non-verbal ability

Non-verbal ability was estimated using the Pattern Construction Subscale of the British Ability Scales-II (Elliott et al., 1996), which measures visuo-spatial ability. Children were requested to copy patterns presented in a book using foam squares (easy), and cubes with patterned sides (hard). The patterns became gradually more complex, and administration was stopped either when a child reached the maximum score, or when they failed to copy four out of five consecutive items. Non-verbal mental age in months was obtained for each child.

#### 2.1.5 Verbal ability

Verbal ability was estimated using the British Picture Vocabulary Scale II (BPVS-II; Lloyd et al., 1997), a measure of receptive vocabulary. Children were presented with four pictures and asked to point to the picture named by the investigator. Stimuli were divided into sets of 12 items. The testing session commenced with presenting items in the basal level appropriate for each age group. Following standardised administration procedures, the items were then presented in a sequence until a child correctly identified 12 subsequent items with no more than one error, following which they moved up to the next level. The task finished if a child made more than eight errors in a given set (8/12 errors).Verbal mental age in months was obtained for each child.

#### 2.1.6 Teacher-rated ADHD symptoms

A measure of ADHD symptoms was obtained from teachers using the Conners Teacher Rating Scale-Revised: Short Version (CTRS-R:S; Conners, 1997)). Teacher ratings were favoured over parental report because we aimed to test the applicability of new our sustained measure to classroom behaviours, with teachers being the most direct informants. This standardised screening scale consists of 28 items that measure indices of oppositional behaviour problems, hyperactive behaviour, cognitive problems, and attention deficits across the school setting in boys and girls aged 3–17. Items were scored on a Likert scale of 0–3. Sub-scales include Cognitive Problems/Inattention, Hyperactivity, Oppositional Behaviour, and an ADHD index (a set of 12 questions based on the Diagnostic and Statistical Manual for Mental Disorders, Fourth Edition; APA, 1994)). The raw scores were standardised based on appropriate age- and gender-matched norm data. The current investigation will only focus on the ADHD index as the dependant variable of interest, for several reasons. First, the Cognitive Problems/Inattention index, which may appear highly relevant as a correlate of sustained attention, comprises items that are more related to academic functioning in the classroom (e.g., ‘not reading up to par’, ‘poor in arithmetic’), and is therefore highly influenced by cognitive problems that may be unrelated to attention. Second, empirical findings have shown that of all the CTRS-R:S factors, the ADHD index has the highest correlation with the standard DSM criteria for ADHD inattention symptoms (e.g., McGoey et al., 2007)). Finally, the ADHD index allows the use of age-appropriate standardised t-scores that may be crucial when comparing behaviour across children of different age, gender, and cognitive impairments, even though it has the inherent limitation of also including symptoms of hyperactivity and impulsivity.

#### 2.1.7 Statistical analysis

To assess group differences in sustained attention, the groups were first compared based on the accuracy rate on the first and the second halves of the task using a repeated-measures ANOVA with the age-group as a between-participants factor. The ANOVA procedure was followed by a more detailed inspection of the performance dynamics for each group separately. We computed mean accuracy on each trial and plotted it as a function of the trial number, thereby constructing a time series. We estimated the linear relationships between trial number and mean accuracy using Pearson's Rho, to determine whether there was any reliable trend of either improvement or decrement in performance over time on task.

Following comparisons across groups via ANOVA, a hierarchical linear regression analysis was conducted to investigate the relationship between ADHD symptoms and performance change. The first group of predictors included the estimated verbal and non-verbal mental age, the second model added group and percentage change in accuracy as a predictor, and the third added the interaction between the two (Group X % Change). The outcome variable was the age-standardised ADHD index based on the CTRS-R:S. Three children (two with Williams syndrome and one with Down's syndrome) had missing CTRS-R:S data and could not be included in this analysis.

## 3 Results

### 3.1 Group comparisons

**Fig. 1:**
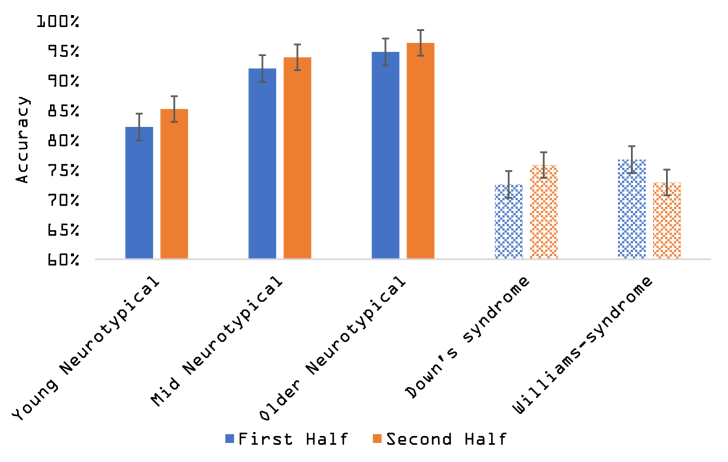
Performance (mean accuracy) of the five groups over two task halves. Blue and orange colours represent the first and second halves, respectively. Full colour bars represent neurotypical populations and the dashed coloured bars represent children with genetic syndromes. (For interpretation of the references to colour in this figure legend, the reader is referred to the Web version of this article.)

Descriptive statistics for performance on the CPT as well as ADHD-Index scores, for the five groups (three neurotypical groups, DS and WS) appear in Table 2. We describe the mean accuracy and the rates of different response types: correct identification of target (‘hit’), and judging a distractor as a target (‘false alarm’). Extracting hit and false-alarm responses allowed us to estimate perceptual parameters based on the Signal Detection Theory (SDT). The SDT perceptual parameters are the target sensitivity (typically signed ‘d-prime’), which stands for the ability to distinguish among targets and distractors and is calculated based on the distance between mean standardised number of ‘hit’ and ‘false alarm’ responses; The second perceptual parameter is the response bias (typically signed‘β’ or criterion), which represents the dominant error type (whether people tend to commit more false alarms, or whether they are more likely to miss targets. Given the small number of trials and number of participants at ceiling, we report an adjusted non-parametric estimation of target sensitivity, A′, and response bias, B’’ (Grier, 1971; Snodgrass and Corwin, 1988; Stanislaw and Todorov, 1999; See equations (1), (2))). Accuracy on the two task halves on each group is illustrated in Fig. 1.

(1) $$A^{'}=.5+\left[\text{sign}\left(H-F\right)\frac{{\left(H-F\right)}^{2}+\left|H-F\right|}{4\max\left(H,F\right)-4HF}\right]$$

**Table 2:** Descriptive Statistics (mean; SD). Abbreviations: ADHD: Attention Deficit/Hyperactive Disorder; SD: Standard Deviation. CPT: Continuous Performance Task. NT: Neurotypical. DS: Down's Syndrome. WS: Williams syndrome.

	ADHD Index	Accuracy	Hit Rate	False Alarms	Sensitivity (A′)	Response bias (B″)
NT-younger (N = 31)	55.16; 11.39	.83; .09	.51; .31	.08; .07	.78; .18	.33;.52
NT-middle (N = 34)	50.62; 10.17	.92; .08	.87; .19	.06; .07	,94; .08	-.03; .77
NT-older (N = 34)	51.71; 11.47	.95; .04	.93; .09	.03; .03	.97; .03	-.32; .73
DS (N = 18)	70.60; 9.57	.74; .13	.63; .24	.22; .24	.77; .17	.27; .48
WS (N = 25)	70.80; 11.06	.75; .18	.36; .23	.16; .16	.66; .16	.31; .34

Equation (1): calculating A’ based on Stanislaw and Todorov, 1999. The letter H stands for Hits, and the letter F stands for False Alarms. When H > F, sign(H-F) is replaced with +1, and in the opposite case is replaced with −1. Max(H,F) equals H or F, whichever is larger.

(2) $$B^{''}=\text{sign}\left(H-F\right)\frac{H\left(1-H\right)-F\left(1-F\right)}{H\left(1-H\right)+F\left(1-F\right)}$$

Equation (2) calculating B″ based on Stanislaw and Todorov, 1999. The letter H stands for Hits, and the letter F stands for False Alarms. When H > F, sign(H-F) is replaced with +1, and in the opposite case replaced with −1.

When comparing the two task halves we focused first on the mean accuracy, and then sought corroborating evidence with a complementary analysis using perceptual sensitivity parameters. The main reason for focusing on mean accuracy is the relatively small number of trials, alongside a high proportion of control participants who did not commit any error (either miss or false alarm) which can affect the reliability of the sensitivity measure (e.g., Miller, 1996; Stanislaw and Todorov, 1999). Group differences in sustained attention were therefore assessed using a repeated-measures ANOVA with the accuracy rate in the first and second halves of the task as a within-subject factor (‘task half’), and the experimental group as a group factor. The test revealed a significant main effect for the group factor (F(4,137) = 22.092; p < .001; Partial η^2^ = 0.392), and no significant effect for the task half factor (p = .09). Crucially, there was a significant interaction between group and the task half (F(4,137) = 3.426; *p* = .013; Partial η^2^ = 0.088).

Further analyses, based on the significant effect of group, were carried out to learn about overall group differences between Williams syndrome, Down's syndrome, and all the other groups. For conciseness, Table 3 lists all the paired comparisons.

**Table 3:** Post hoc paired comparisons of overall performance between groups, corrected for multiple comparisons. Abbreviation: DS – Down's Syndrome; WS – Williams-syndrome. *p < .01; **p < .001.

Group A	Group B	Mean Difference	95% CI (Lower Bound)	95% CI (Upper Bound)
WS	DS	0.006	−0.060	0.073
	Young Neurotypical	-.089^*^	−0.147	−0.031
	Mid Neurotypical	-.177^**^	−0.233	−0.120
	Oldest Neurotypical	-.207^**^	−0.264	−0.151
DS	Young Neurotypical	-.096^*^	−0.159	−0.032
	Mid Neurotypical	-.183^**^	−0.246	−0.121
	Oldest Neurotypical	-.213^**^	−0.276	−0.151

The comparisons table shows that children with Down's syndrome and children with Williams syndrome performed worse than all neurotypical groups, including much younger children of comparable verbal and non-verbal ability. Overall performance for children with Williams syndrome and children with Down's syndrome was equivalent. As shown in Fig. 1, descriptively, all groups but the Williams syndrome had a higher accuracy on the second half of the task when compared with the first half. This effect is also likely to underlie the observed interaction, which was further analysed. A *post hoc* planned comparison confirmed that there was a significant decrement in performance between the two halves of the task in the WS group (t(24) = 2.268; p = .033; 95% CI [0.003 0.073]), with a better performance in the first half (mean accuracy = 76%; SE = 17%) compared to the second half (mean accuracy = 72%; SE = 19%). When repeating the same comparison in all other groups, there were no significant differences between the two halves in the group of children with DS (p = .27) and the groups of oldest (p = .215) and mid neurotypical (p = .077). The youngest group of neurotypical children showed a pattern of improvement between halves (t(29) = --2.388; p = .024; 95% CI [-0.060;-0.004]) As indicated by the significant interaction in the ANOVA analysis, followed by the posthoc t-tests, that there was a pattern of a decrement in performance over time only for the Williams syndrome group.

For completeness, we repeated a portion of the analysis procedures described hitherto with perceptual sensitivity (A′) as the dependent variable. Although estimating perceptual sensitivity based on a small number of trials and high proportion of ceiling performers could impair its reliability (Miller, 1996; Stanislaw and Todorov, 1999), it is important to test whether we can find corroborating evidence on this additional index of performance. We used a repeated-measures ANOVA with perceptual sensitivity (A′) in the first and second halves of the task as a within-subject factor (‘task half’), and the experimental group as a group factor. The test revealed a significant main effect for the group factor (F(4,137) = 23.235; p < .001; Partial η^2^ = 0.404), and no significant effect for the task half factor (p = .54). A series of post-hoc group comparisons, corrected for multiple comparisons, indicated that the main effect of group depended on the following group differences: the WS and the DS groups did not differ in their overall A’ (p = .348); both groups had overall lower perceptual sensitivity compared to the oldest- and mid-neurotypical (all p's < 0.001); and the WS group also performed poorer than the youngest control group (p = .024) while the DS group did not (p = .342). There was a significant interaction between group and the task half (F(4,137) = 3.121; *p* = .017; Partial η^2^ = 0.084). Convergent with the analyses on accuracy, a further analysis confirmed that there was a significant decrement in A′ between the two halves only in the group of children with WS (t(24) = 2.156; p = .041; 95%CI[0.002;. 111]).

### 3.2 Continuous performance data: time-series analysis

To present a faithful description of the performance dynamics on each group, we carried a secondary analysis where we investigated mean accuracy on each trial. The continuous data series was then fitted to a linear trend and was estimated using a Pearson correlation test between trial number and mean accuracy. A negative correlation between the trial number and mean accuracy would provide further support to the notion of performance decrement in children with WS as reported based on the ANOVA test. We present a full description of the linear trend, the mean accuracy on each trial, and the mean accuracy based on a moving average window of ten trials. Pearson's Rho values and their associated p-values are overlaid on the charts.

**Fig. 2:**
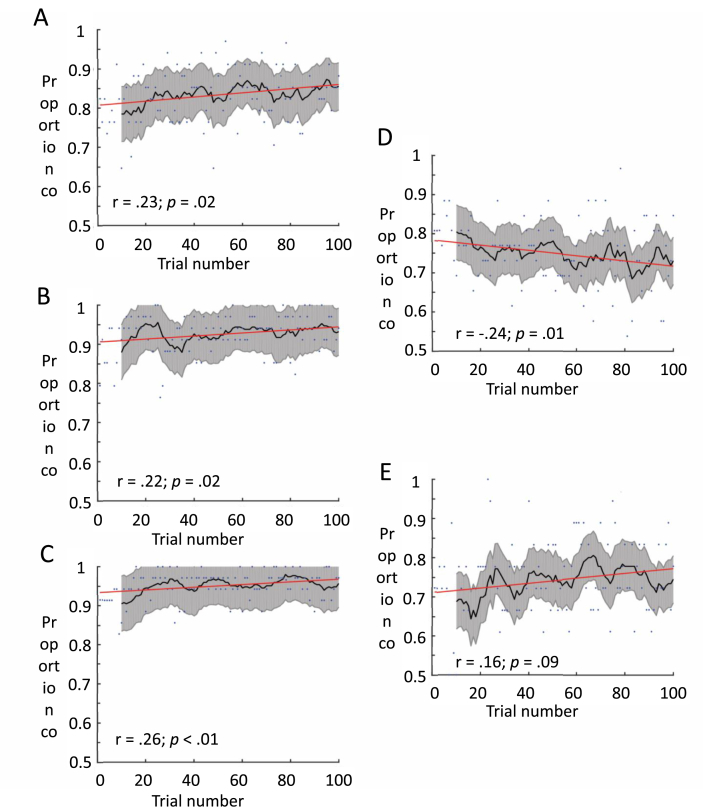
Course of change in performance (mean accuracy) over time on task. Blue dots represent group mean accuracy on each trial. Red lines represent the linear fit. Black lines represent the moving average window, averaging performance over ten trials at each point. Shaded grey colours represent the standard error of the average moving window. Pearson's Rho values and their associated p-vales appear on each figure. Groups: A) young neurotypical; B) Mid Neurotypical; C) Older Neurotypical; D) Children with Williams syndrome; E) Children with Down's syndrome. (For interpretation of the references to colour in this figure legend, the reader is referred to the Web version of this article.)

As represented in Fig. 2, when inspecting the continuous data by plotting mean accuracy at each time point during the task, we revealed a linear trend of increase in accuracy as a function of trial number in the three control groups (panels A–C). An opposite trend appeared in the group of children with Williams syndrome, where there was a negative correlation between trial number and accuracy (Fig. 2, panel D). Finally, there was no significant trend of change in performance when inspecting the data of children with Down's syndrome.

#### 3.2.1 Task performance and inattention symptoms

After focusing on group differences in the previous section, the next analysis tested how performance decrement (accuracy-change) related to ADHD symptoms. Everyday problems in attention in children are typically estimated by using a standardised questionnaire designed to assess behavioural and cognitive problems by focusing on items related to ADHD. To learn about the relations, a regression analysis was applied to the age-appropriate standardised ADHD symptom scores (reported by the teachers) as the dependent variable, and mental age and performance change as predictors. The use of standardised ADHD scores controlled for age-related differences.

A hierarchical regression model was applied to control for differences that can be explained by verbal and non-verbal mental age. The reason for controlling the mental-age variables is to account for symptomatic differences that are related to a ‘mismatch’ between chronological age and developmental level for children with WS or DS. The logic of using age-standardised scores as the dependent variable relies on the assumption that inattention and hyperactive/impulsive behaviour varies with age. Accordingly, it is important to account for age and ability differences that are not accounted for within the normative standardised attention scores. To do so, the first block of predictors included verbal and non-verbal mental-age in months. In the second block of predictors, the accuracy-change and group factors were added (defined as the percentage change in accuracy between the first and second task halves). A third block of predictor also included the interaction between group and accuracy-change factors. The regression model and its results appear in Table 4. The third model, which included the interaction between group and % change in accuracy, did not change fit significantly.

**Table 4:** Regression table, describing the two models and the change statistics. *p < .05; **p < .001; The third model, which included the interaction between group and % change in accuracy, did not change significantly.

	β	R^2^	R^2^ Change	F Change
**Model #1**		.117	.117	13.819**
Verbal Mental Age	.150			
Non-Verbal Mental Age	-.474**			
**Model #2**		.150	.034	6.586*
Verbal Mental Age	.134			
Non-Verbal Mental Age	-.399**			
% Change in Accuracy	-.157*			
Group	208*			
**Model #3**			.345	N.S (p > .5)

As shown in Table 4, non-verbal mental age significantly predicted ADHD symptoms, whereas verbal mental age did not, accounting for 12% of the variance in ADHD symptoms. The second model, which incorporates the proposed accuracy-change index, increased the explained variability of the model by 3%, and supported a significant contribution of the sustained attention change measure. The negative relationship suggests that ADHD symptoms were more severe as the decrement in performance on the CPT increased.

## 4 Discussion

This study presents a novel experimental approach in WS and DS, emphasising the notion of change in performance over time. Children with Down's syndrome and Williams syndrome underperformed overall compared to younger neurotypically developing children and had overall comparable detection rate on the CPT. These findings resemble previous reports of an overall impairment for both groups (e.g., Brown et al., 2003; Costanzo et al., 2013; Rowe et al., 2006). Nevertheless, here we show that the two groups differed in their ability to sustain performance over time. A significant decrement in performance was observed only within the group of children with Williams syndrome. Accordingly, we propose that the WS group are more likely to suffer from a selective impairment in the ability to sustained attention over time, whereas children with DS have a more general performance difficulty. To validate that the change in performance over time for children with WS was related to sustained attention in every day settings, a multiple-regression analysis was applied with ADHD symptoms as the dependent variable and the percentage change in accuracy as a regressor (controlling for mental-age indices). In accordance with the research hypothesis, the results demonstrated that percentage change in performance contributed significantly to the regression model, explaining added variance in teacher reports of ADHD symptoms.

These findings also suggest that symptoms of ADHD are more closely related to tasks involving visuo-spatial abilities, as reflected in the two levels of the regression analysis, than to verbal ability: the ADHD index negatively correlated with non-verbal mental age, and negatively correlated with the percentage change in performance. There was no association between the ADHD index and verbal mental-age, a finding that resembles previous reports in children with WS and DS (e.g., Cornish et al., 2012) Although ADHD is often associated with poor academic skills, including verbal skills, such a difficulty is considered to be secondary, with a late onset (e.g., Ek et al., 2011). Of note, here, verbal mental age was also assessed using a comprehension task, a language skill less likely to be impaired in ADHD (Andreou et al., 2005). Non-verbal skills, on the other hand, are thought to be closely related to attention (e.g., Duncan, 1995; Duncan et al., 1995), and arguably there are strong associations between sustained attention capacity and non-verbal measures of intelligence (Stankov, 1988).

This study addressed a major concern in neuropsychological assessment: while clinical populations often exhibit lower performance than controls in cognitive tasks, their performance pattern is not necessarily driven by comparable cognitive factors. To reveal the processes underlying group differences, it is necessary to extract performance indices that go beyond average performance. Specifically, we argue that, although both children with Williams syndrome and Down's syndrome may present poorer performance on CPTs when compared with age-matched controls, it does not follow that both groups suffer from poor sustained attention. While the CPT was originally designed to measure sustained attention, not all task indices reflect the construct of interest: in relying solely on general accuracy, researchers abandon the temporal element, which is key to understanding sustained attention. Keeping in mind this critical view, it is of note that, to our knowledge, this is the first attempt to assess a *decrement* in performance, rather than *overall* poor performance, in children with Williams syndrome or Down's syndrome.

Our findings suggest that children with Williams syndrome have difficulty in maintaining attention, as reflected in a significant accuracy decrement over the second half of the task. In contrast, children with Down's syndrome did not show sign of performance decrement. Instead, their pattern of performance over time was overall poorer than that of control children, but similar to that of all control groups, with overall higher accuracy rate over the second half. Our observations may have direct implications on educational approaches applied when working with WS children. The evident group effect of performance decrement while engaged in a simple task can inform educators designing class activities and content. Future interventional studies can explore the potential benefits of frequent breaks to improved engagement over time.

Of note, a limitation emerged specifically with regards to children with DS, and one that could not be solved even with our novel analytical approach: given that a significant proportion of children with DS (7 children, 25%) did not complete the task, our findings of poor performance in this group may remain an overestimation of the general sustained attention skills in this group. Researchers may need to focus on different assessment methods better tailored to engage all children with DS.

In conclusion, we tackled a common problem of the current literature reporting sustained attention difficulties across atypically developing populations: overall poor performance on classic attention tasks such as the continuous performance task may mask distinct profiles of performance over the duration of assessment. Here, these dynamic changes distinguished young children with WS from children with DS, despite their overall similarly poor performance. In addition, the dynamic change measure predicted teacher-reported ADHD symptoms. This approach may be fruitful in characterising sustained attention more clearly in young children with atypical development, for whom more sophisticated measures of sustained attention may be too challenging.

## Footnotes

2 Mosaicism in Down's syndrome means that some cells of the body have trisomy 21, and some have the typical number of chromosomes. This contrasts with the standard presentation of Down's syndrome, in which all cells contain an extra copy of chromosome 21.
